# First identification of the benzimidazole resistance-associated F200Y SNP in the beta-tubulin gene in *Ascaris lumbricoides*

**DOI:** 10.1371/journal.pone.0224108

**Published:** 2019-10-17

**Authors:** Luis Fernando Viana Furtado, Celi da Silva Medeiros, Luciana Werneck Zuccherato, William Pereira Alves, Valéria Nayara Gomes Mendes de Oliveira, Vivian Jordania da Silva, Guilherme Silva Miranda, Ricardo Toshio Fujiwara, Élida Mara Leite Rabelo

**Affiliations:** Departamento de Parasitologia, Universidade Federal de Minas Gerais, Belo Horizonte, Minas Gerais, Brazil; Imperial College London, UNITED KINGDOM

## Abstract

The main control strategy for *Ascaris lumbricoides* is mass drug administration (especially with benzimidazoles), which can select strains of parasites resistant to treatment. Mutations in the beta-tubulin isotype-1 gene at codons 167, 198 and 200 have been linked to benzimidazole resistance in several nematodes. The mutation in codon 200 is the most frequent in different species of parasites, as previously observed in *Necator americanus* and *Trichuris trichiura*; however, this mutation has never been found in populations of *A*. *lumbricoides*. This study aimed to screen for single nucleotide polymorphisms (SNPs) in the beta-tubulin isotype-1 gene at codon 200 in *A*. *lumbricoides*. We developed a technique based on an amplification refractory mutation system (ARMS-PCR) for the analysis of 854 single *A*. *lumbricoides* eggs collected from 68 human stool samples from seven Brazilian states. We detected the mutation in codon 200 at a frequency of 0.5% (4/854). This is the first report of this mutation in *A*. *lumbricoides*. Although the observed frequency is low, its presence indicates that these parasite populations have the potential to develop high levels of resistance in the future. The methodology proposed here provides a powerful tool to screen for the emergence of anthelmintic resistance mutations in parasitic nematode populations.

## Introduction

*Ascaris lumbricoides*, *Trichuris trichiura* and hookworms are soil-transmitted helminths (STH) closely related to precarious living conditions. More than a quarter of the world's population is at risk of infection with these nematodes, which can cause serious damage to human health [1]. Among these helminths, *A*. *lumbricoides* usually has the highest prevalence, infecting 820 million people worldwide, especially in developing countries [2]. *A*. *lumbricoides* may cause an asymptomatic infection or lead to different clinical manifestations, such as acute abdomen, so that the mortality accounts for approximately one-sixth of the disease burden [3].

According to the World Health Organization [1], mass drug administration (MDA) is the main strategy to control the continued occurrence of STH infection, especially with benzimidazole drugs (such as albendazole and mebendazole). The periodic treatment with these drugs can potentially select subpopulations of parasites that become resistant to drug [4–6].Single nucleotide polymorphisms (SNPs) in the beta-tubulin isotype 1 gene at codons 167 –F167Y (TTC, TTT/Phenylalanine → TAC, TAT/Tyrosine), 198 –E198A (GAG, GAA/Glutamic acid → GCG, GCA/Alanine) and 200 –F200Y (TTC/Phenylalanine → TAC/Tyrosine) have been linked to benzimidazole resistance in helminths [7,8] and fungi [9]. In *A*. *lumbricoides*, a mutation at codon 167 was detected at high frequencies in Haiti, Kenya, and Panama in populations that were treated with benzimidazoles [10]; however, mutations at codons 198 or 200 have never been found in this parasite, even though it has already been investigated [7,8,11]. In veterinary ascarids, such as *Parascaris equorum* and *Ascaris galli*, these mutations have never been found, even in populations that were constantly treated with benzimidazoles [12–14].

In Brazil, only a few studies have analyzed the efficacy of drugs in the treatment of *A*. *lumbricoides* [11,15]. In the northeast of Brazil, it has been reported that the improvement of sanitary conditions of the population was not sufficient to eliminate the presence of *A*. *lumbricoides* [15], with reports of persistence of infection even after MDA administration being performed three times a year [16]. In our previous study, mutations were not found in codons 167 and 198 of *A*. *lumbricoides* in samples from Brazil [11]. As the mutation in codon 200 of *A*. *lumbricoides* does not create or eliminate any restriction enzyme cleavage site, making it impossible to use the RFLP-PCR technique, this codon was not included in the initial analyzes. Based on the absence of mutations in the two codons initially analyzed, the aim of the present study was to standardize a molecular tool based on an amplification refractory mutation system (ARMS-PCR) and perform a screen for the F200Y SNP in the beta-tubulin isotype-1 gene in seven *A*. *lumbricoides* populations. We did not have information on the population’s treatment history or on the possibility of drug resistance. However, at low frequency, our data revealed for the first time the presence of the mutation in codon 200 in this species, which does not suggest a current resistance problem, but may indicate that these parasites have the potential to become resistant.

## Methods

### Ethics approval

This work was approved by the Comitê de Ética em Pesquisa–COEP (CAAE 61047216.7.0000.5149) from Universidade Federal de Minas Gerais (UFMG). As we used human feces obtained from commercial laboratories performing pathological analyses, an informed consent document was not required. We did not obtain any subject identification, and the data were analyzed anonymously.

### Sample processing

Stool samples were processed, and DNA extractions were performed from 854 single *A*. *lumbricoides* eggs from 68 patients collected in seven Brazilian states, exactly as described previously [11]. Human coproparasitological collection and screening analysis were performed in seven Brazilian states. Positive samples for *A*. *lumbricoides* were stored in 10% formaldehyde for later molecular analysis. In summary, 2.0 ml of stool suspension was homogenized, filtered through gauze and transferred to a 15ml tube. Five ml of sulfuric ether was added to the suspension and then stirred vigorously, followed by 1 minute centrifugation at 14,000 x g. The supernatant was discarded. Eggs were washed in a new step by adding 500 μl 5.0% of hypochlorite for 10 minutes to the samples. The material was centrifuged at 14,000 x g, and the supernatant was discarded. The eggs were washed again using 500 μl of ultrapure water, followed by centrifugation at 14,000 x g. The supernatant was then discarded and the following steps were performed: a) incubation at 30°C in 500 μl of 0.2 N sulphuric acid for 30 days (for larvae development), b) centrifugation at 14,000 x g and discard of the supernatant, c) washing (resuspension in 500 μl of ultrapure water, centrifugation at 14,000 x g and discard of the supernatant), d) incubation with 500 μl of 1.0% hypochlorite up to the point at which the outerlimiting membrane dissolved using a microscope for visual confirmation, e) repetition of steps a-c followed by the addition of 100 μl of ultrapure water. For DNA extraction, the eggs were observed under an optical microscope, individually pipetted into a volume of 1 μl and transferred to a 500 μl microcentrifuge tube containing 10 μl of buffer, as described by Diawara and colleagues [7]. Table 1 shows the data relating to the sample number.

**Table 1:** Collection sites, number of patients and eggs of *A*. *lumbricoides* used for drug resistance-related SNP genotyping.

	Patients	Total eggs	Eggs per patient (Minimum and maximum)
**Bahia**	7	75	10–16
**Ceará**	16	185	9–14
**Maranhão**	13	158	8–18
**Minas Gerais**	7	94	10–19
**Piauí**	10	123	10–16
**Sergipe**	3	57	16–20
**Tocantins**	12	162	11–16
**Total**	**68**	**854**	

### Primer design

Primers were designed using Primer3 (http://bioinfo.ut.ee/primer3-0.4.0/), which were based on the beta-tubulin isotype-1 sequence from *A*. *lumbricoides* available in the Genbank database (http://www.ncbi.nlm.nih.gov/genbank/) under the accession number FJ501301.1. According to the purpose of the technique, changes were made in the base sequences of some primers (explained below). Table 2 shows all the primers designed in this study.

**Table 2:** Primers used, their respective annealing temperatures and positions of the substitutions (when applicable). The positions where changes have been made are underlined.

Primer (5’– 3’)	Change
*AltubR*: GGT TGA GGT CTC CGT ATG TG	
*AltubF*: ATG TGA GAA AAT GCG GTC AT	
*F200mut*: ACC GAT GAA ACC T**A**C TGC AT	T→A
*Fc200Al*: GGC AGC TGA ATG GAG AGC	
*Fs200 Al*: TGA GAA CAC CGA TGA AA**G** CTT	C→G
*Rr200Al*: CAA AGC CTC ATT GTC AAT G**G**A GT	C→G
*Rc200Al*: CTC CGT ATG TGG GAT TTG TAA GC	

### Synthesis of recombinant plasmids for control

For the standardization of the technique and for use as controls in reactions for analysis of codon 200, plasmids were synthesized without mutation (wild-type) and with the mutation of this codon. Since codons 167, 198 and 200 in the beta-tubulin isotype-1 gene of *A*. *lumbricoides* are close to each other in the genome, the wild-type control of codon 200 was the same as that synthesized for the other two codons by Zuccherato and colleagues [11]. All PCR amplifications in this study were performed using GoTaq Green Master Mix (Promega, USA), with a final concentration of 0.2 μM for each primer, according to the following program: 95°C for 5 min, 30 cycles at 95°C for 30 s, 60°C for 45 s, 72°C for 60 s and a final step of 72°C for 7 min. A “blank” sample was included in all amplification runs in which the DNA was replaced with water to assess the presence of possible contaminants.

**Fig 1:**
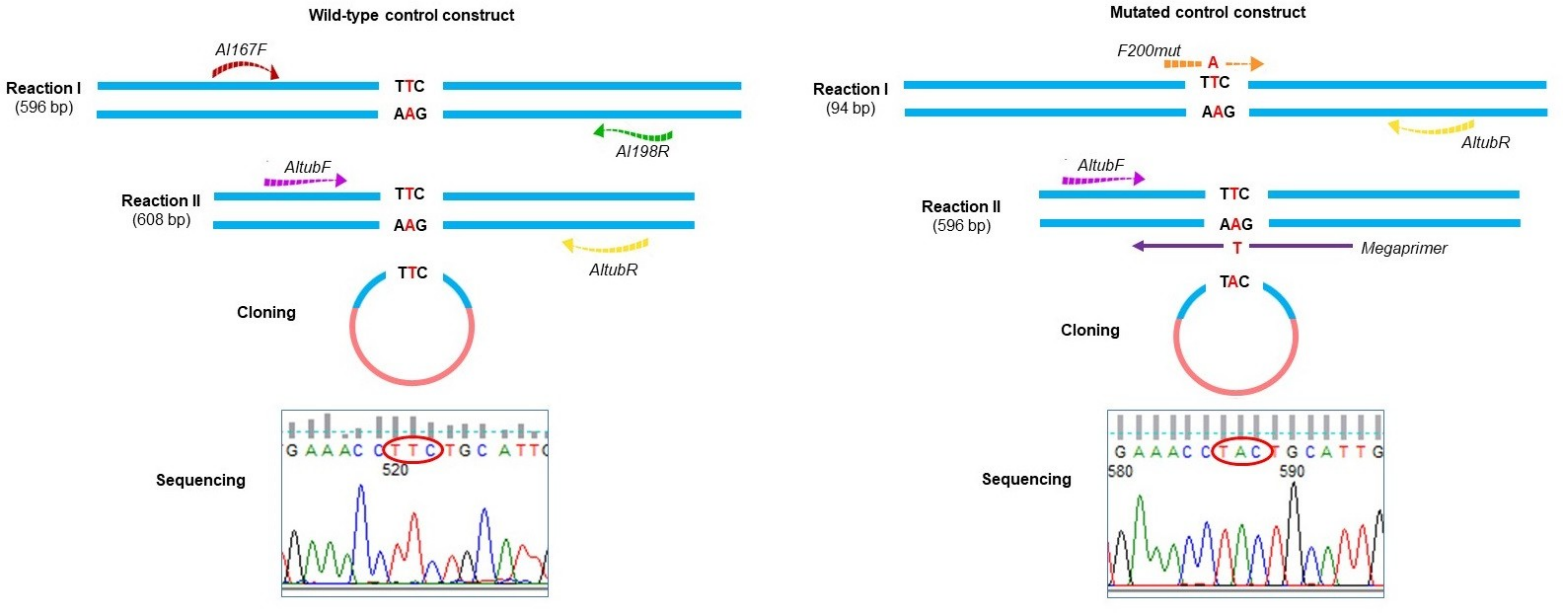
The scheme adopted for the construction of the controls without mutation (wild type) and with the mutation for the analysis of codon 200 in the beta-tubulin gene of *A*. *lumbricoides*. The wild-type control was constructed by Zuccherato and colleagues [11].

To synthesize the mutated control plasmid, site-directed mutagenesis was performed using the Megaprimer-PCR technique, as described by Furtado and Rabelo [17] for *Ancylostoma caninum*. The wild-type control was employed as a template for PCR amplification using the primer combination of *F200mut* + *AltubR* (94 base pairs, bp). The *F200mut* primer was designed to include a mismatch at position 7 of the 3ꞌ-end of the primer that replaced a T nucleotide with an A to mimic the mutated sequence. The reaction product was subjected to electrophoresis on a 1.0% agarose gel (w/v) (Midsci, St. Louis, USA) with 0.5x Tris-acetate EDTA (TAE) buffer, and the gel was stained with GelRed (Biotium, USA). The fragment was then excised from the gel and purified (Illustra GFX PCR DNA and Gel Band Purification Kit, GE Healthcare, UK), and the concentration was determined. Approximately 25.0 ng of the first reaction product was used as a forward megaprimer in the second reaction, in combination with 1 μM final concentration of the *AltubF* primer (596 bp). The fragment was subsequently cloned using the pGEM-T Easy Vector System (Promega, USA), transformed into XL1-blue cells (Phoneutria, Brazil) and recovered via miniprep (Wizard Plus Miniprep DNA Purification System, Promega, USA). The plasmid was sequenced, and the presence of the mutation was successfully confirmed. Fig 1 illustrates the scheme adopted for the synthesis of controls.

### Genotyping of single *A*. *lumbricoides* eggs

For the analysis of codon 200 in the beta-tubulin isotype-1 gene of *A*. *lumbricoides*, a technique based on an amplification refractory mutation system (ARMS-PCR) was adopted. The PCRs for these analyses were performed under the same conditions described previously (in the section “Synthesis of recombinant plasmids for control”). A first PCR amplification with the primers *AltubF* + *AltubR* (629 bp) was performed for each sample. From this first reaction, two independent nested PCRs were performed. One reaction was performed using the primers to detect the wild-type allele (*Fs200Al* + *Rc200Al* = 92 bp), and another reaction was performed using the primers to detect the polymorphic allele (*Fc200Al* + *Rr200Al* = 181 bp).

**Fig 2:**
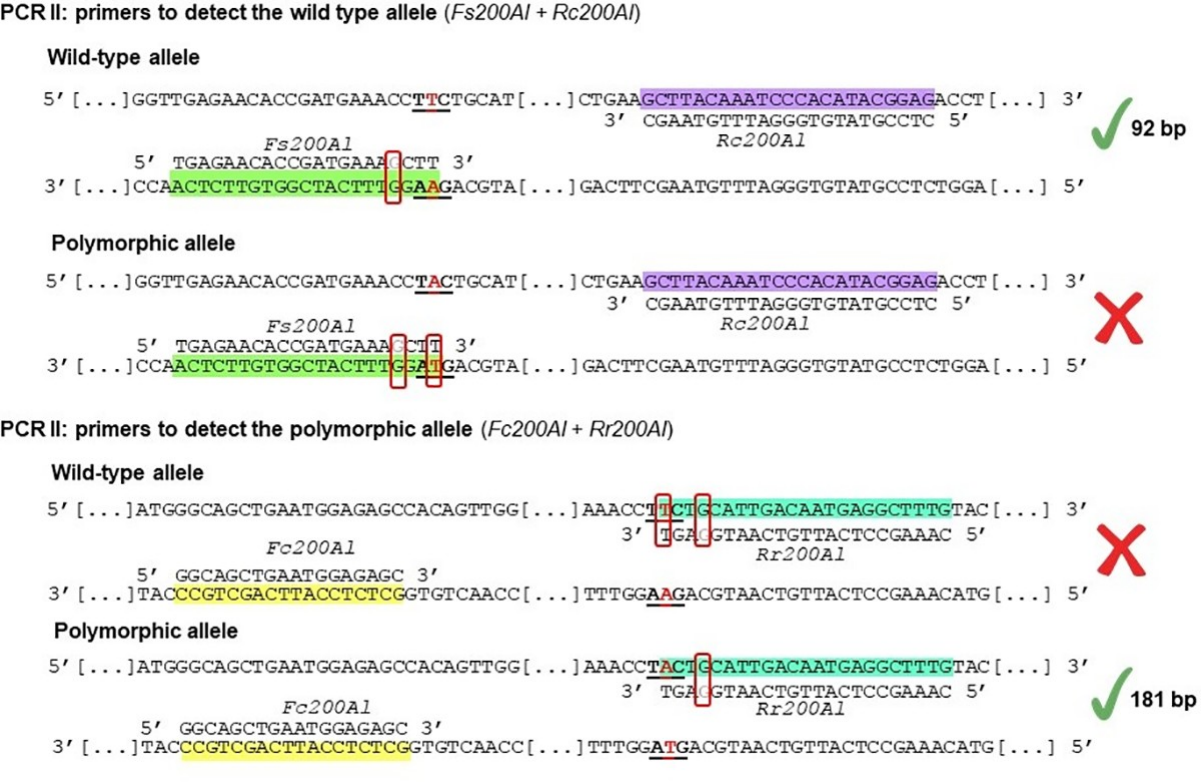
Representation of the ARMS-PCR strategy used for analysis of beta-tubulin codon 200 of *A*. *lumbricoides*. Analyses were performed by ARMS-PCR in two individual reactions. The *Fs200Al* primer was designed to anneal only in the absence of the mutation, whereas the *Rr200Al* primer was designed to anneal in the presence of the mutation. The annealing sites of the *Fs200Al*, *Rc200Al*, *Fc200Al* and *Rr200Al* primers are marked with green, purple, yellow and blue, respectively; codon 200 is underlined, with the base of interest in red. In the primers, the bases marked in gray correspond to the location of the second incompatibility, and the boxes in red represent the incompatibilities in each sequence.

The *Fs200Al* primer was designed to anneal only to TTC (wild-type allele), while the *Rr200Al* primer was designed to anneal only to TAC (polymorphic allele). Both primers had a mismatch at position 4 at the 3′ end to ensure high specificity, as described in other studies [18–20]. The products of these reactions were subjected to electrophoresis in 2.0% agarose gels (w/v) (Midsci, USA) with 0.5x TAE buffer, and the gels were stained with GelRed^™^ (Biotium, USA). Fig 2 shows a representation of the ARMS-PCR used for analysis of beta-tubulin isotype-1 codon 200 of *A*. *lumbricoides*. In cases of samples with mutated alleles, sequencing was performed for confirmation. For the validation of the results obtained in the ARMS-PCR were sequenced 52 samples of single eggs. Sequencing was performed according to the method originally described by Sanger and colleagues [21]. For this, from the first PCR product (*AltubF* + *AltubR* = 629 bp) a nested PCR was performed with the primers *Fc200Al* + *Rc200Al* (230 bp), under the same conditions described above. The reaction product was subjected to electrophoresis on a 1.0% agarose gel (w/v) (Midsci, St. Louis, USA) with 0.5x TAE buffer, and the gel was stained with GelRed (Biotium, USA). The fragment was then excised from the gel and purified (Illustra GFX PCR DNA and Gel Band Purification Kit, GE Healthcare, UK), and the concentration was determined. Sequencing reactions were performed using the BigDye Terminator v3.1 Cycle Sequencing Kit (Applied Biosystems, USA) on the ABI 3130x1 / Genetic Analizer automated sequencer (Applied Biosystems, USA). Each sample was sequenced forward and reverse, and chromatogram analysis was performed using FinchTV software (Geospiza, USA). Sequences of mutated and non-mutated samples were deposited in the Genbank database under accession number MN460676 and MN460677, respectively.

## Results

**Fig 3:**
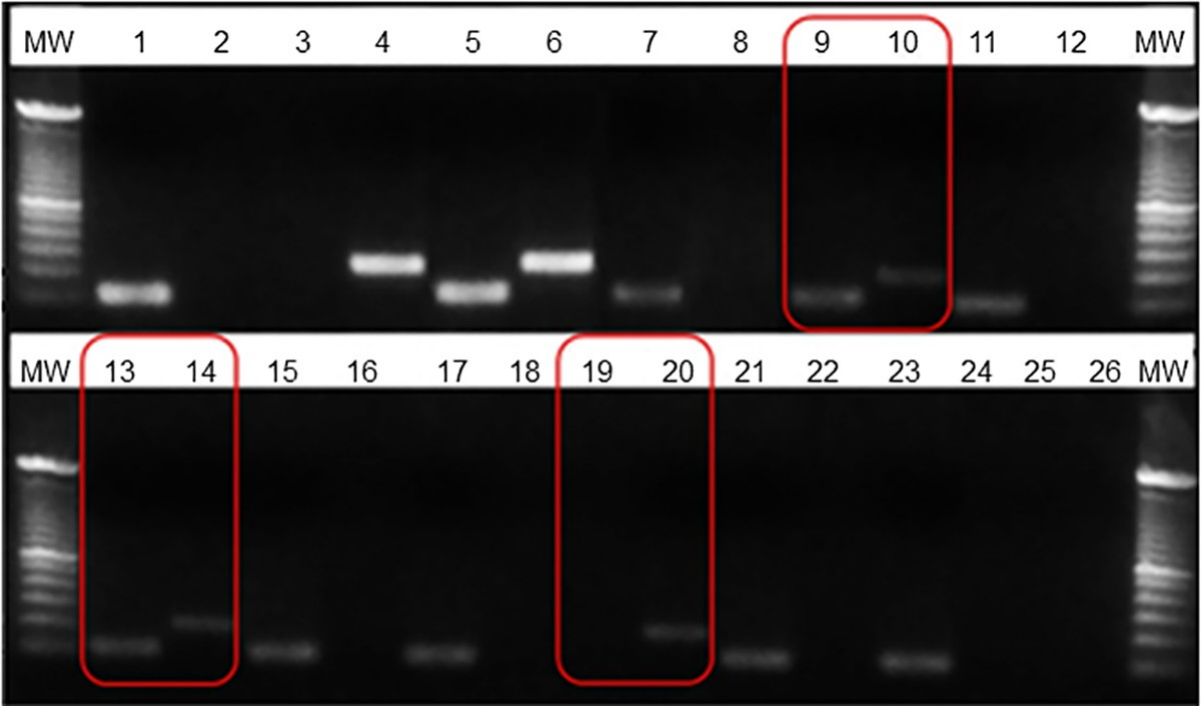
Representative ARMS-PCR results from the screening in the beta-tubulin gene at codon 200 in *A*. *lumbricoides*. Genomic DNA samples from individual eggs were subjected to ARMS-PCR amplification with the two sets of primers. Lanes indicated by odd numbers correspond to ARMS-PCR products obtained using the primer combination *Fs200Al* + *Rc200Al* to detect the fragment without the mutation (92 bp), and the even-numbered lanes correspond to ARMS-PCR products obtained using the primer combination *Fc200Al* + *Rr200Al* to detect the fragment with the mutation (181 bp). In lanes 1–6, synthesized controls were used (lanes 1 and 2: wild-type plasmid; lanes 3 and 4: mutated plasmid; lanes 5 and 6: wild-type and mutated plasmid mix. For each sample, the two reactions were analyzed side-by-side. Products marked in red represent mutated samples. The image shows an agarose gel (2.0%) that was stained with GelRed^™^ (Biotium, USA). MW: 100-bp molecular weight.

We analyzed 854 single *A*. *lumbricoides* eggs from 68 patients collected in seven Brazilian states. Of this total, a mutation at codon 200 in the beta-tubulin isotype-1 gene was observed in 0.5% (4/854) of the eggs. Two geographic locations presented mutations: 1) Maranhão, with 0.6% positivity (1/158): one homozygous egg from a patient with 14 eggs analyzed. 2) Minas Gerais, with 3.2% positivity (3/94): one homozygous and two heterozygous eggs from the same patient (12 eggs analyzed). The mutated samples were sequenced, and the presence of the mutation was successfully confirmed. Sequencing of the other 52 randomly selected samples validated the standardized molecular technique. S1 Fig shows chromatograms referring to the sequencing of the mutated samples. Fig 3 shows a representative agarose gel image of both ARMS-PCR products.

## Discussion

MDA has been used as an effective means of reducing morbidity from helminths, limiting transmission within endemic communities. Although this strategy has benefits for the population, some undesirable effects may occur, such as the reduction in the effectiveness of treatment [22,23]. This reduction in treatment effectiveness occurs in a worrisome manner in veterinary parasites (as in *Haemonchus contortus* [5,6]), with the establishment of nematodes that present high levels of resistance to drugs such as benzimidazoles. SNPs in the beta-tubulin isotype 1 gene have been linked to benzimidazole resistance in these helminths [4–6]. Although the anthelmintic resistance problem is not as clear in human health, some of these SNPs have already been described for STHs in some parts of the world [7,10,11]. Here, we screened for the mutation in codon 200 of individual *A*. *lumbricoides* eggs collected from feces of individuals from seven Brazilian states.

Generally, while the efficacy of benzimidazoles against *T*. *trichiura* is considered low [24], efficacy against hookworms and *A*. *lumbricoides* is high (with cure rate greater than 90%) [25]. However, there are reports of failure to treat *A*. *lumbricoides* in different locations around the world [26–28]. Mutation at codon 167 was detected in *A*. *lumbricoides* [10]; but mutations at codons 198 or 200 have never been found in this parasite [7,8,11]. Rashwan and colleagues [8] and Diawara and colleagues [10] studied the codon 200 in *A*. *lumbricoides* collected from Haiti and Panama. Diawara and colleagues [7] analyzed samples from Kenya; however, none of these studies detected alterations at codon 200 in the beta-tubulin isotype-1. In our study, 0.5% of the 854 single eggs showed mutations at codon 200. To our knowledge, this is the first report of this mutation in parasites of this species.

The prevalence observed in our results is lower than the prevalence reported for veterinary parasites, as in *H*. *contortus* [29,30]. However, in some studies that analyzed these SNPs in ascarids of veterinary importance (i.e *P*. *equorum* and *A*. *galli*) [12–14], these changes were not found, even in parasite populations that were treated many times a year. This suggests that these SNPs may not correspond to the main mechanism of benzimidazole resistance in these species. We suggest that experimental selection of an *A*. *galli* resistant strain by selective drug pressure, using birds for infection, may help elucidate these mechanisms, just as our group performed with *Ancylostoma ceylanicum* in hamsters [31].

The major limitation of our study was not having access to the medical records of the patients analyzed. In view of the low frequency of mutations found, it is likely that these patients were not submitted to MDA treatment, or if they did receive treatment, the periodicity of the MDA was not sufficient to confer a high frequency of the mutated allele. These hypotheses are consistent with the results obtained previously by our group when we evaluated codons 167 and 198 of some of these same samples [11]. If these populations had been subjected to frequent treatments, probably SNPs linked to benzimidazole resistance would be more frequent, as described for other parasite populations [29,30].

To detect mutations related to resistance to benzimidazoles, some tests have been proposed for helminths [10,31] and fungi [9]. In this study, a molecular test was developed to detect mutations at codon 200 in the beta-tubulin isotype-1 gene of *A*. *lumbricoides*. Mutated and wild-type controls were synthesized to standardize the reactions, and controls previously developed for this purpose in the study of other helminths were also used [31,32].

Our analyses were performed by ARMS-PCR, with two reactions for each sample, one reaction using a primer pair to detect the wild-type fragment and another reaction with a primer pair to detect the mutated fragment. For the implementation of this methodology, a second sequence incompatibility was added at the position 4 at the 3' end of the primer designed to anneal only in the presence of the mutation (*Fs200Al*) or in the absence of the mutation (*Rr200Al*). However, some authors, such as Albonico and colleagues [33], reported not needing to add this incompatibility to analyze codon 200 of *N*. *americanus*. The addition of this second incompatibility may be necessary to guarantee the specific annealing of the primer in question, as found by Furtado and Rabelo [18] in the analysis of codon 200 of *A*. *caninum*. These authors pointed out that the absence of this second incompatibility made the primer nonspecific, annealing in both, in the presence and in the absence of the mutation, suggesting that the need for the addition is related to the composition of the target sequence itself.

For our analyses, the tetra-primer PCR technique was tested with the four primers in the same reaction, but the results were not satisfactory, with the appearance of many nonspecific amplicons for both, the recombinant plasmids and the genomic DNA. The standardization of this methodology can be laborious due to the complexity of the functioning of the four primers in the same reaction, being able to produce more fragments than expected since there is no flexibility to alter the sequence of the primers because the region in which the primers anneal is determined by the SNP site, and it is thus, only possible to change the primer length [19]. Tetra-primer PCR has previously been applied for the analysis of SNPs linked to resistance in other helminths [20,34].

Studies based on real-time PCR (qPCR) and sequencing have previously been described in the literature for the analysis of SNPs in helminths [6,32]. Diawara and colleagues [10] evaluated SNPs in the beta-tubulin isotype-1 gene of *A*. *lumbricoides* by pyrosequencing, emphasizing that the technique is very sensitive for this purpose. However, pyrosequencing requires specific equipment, whereas ARMS-PCR requires the use of only a conventional thermocycler, providing a direct result without the need for graphical analysis or chromatograms.

Rashwan and colleagues [8] developed an SNP genotyping assay for *A*. *lumbricoides* by the SmartAmp2 method; however, the combination of several initiators (from five to six) in the same reaction may hinder the proper functioning of this method. Furtado and colleagues [31] analyzed mutations in the beta-tubulin isotype-1 gene of *Ancylostoma braziliense* by restriction fragment length polymorphism (RFLP-PCR); other authors have also used this method for studies of several other nematodes [11,29,35]. Although RFLP-PCR is considered simple and sensitive, it has some limitations, such as in cases where the DNA sequences are not recognized by commercial restriction enzymes or harbor many recognition sites for a single enzyme [36]. In addition, many restriction enzymes have a high financial cost. Baltrušis and colleagues [37] analyzed the beta-tubulin isotype 1 gene of *H*. *contortus* by the Next-generation sequencing (NGS) technique, which has the great differential of providing direct and parallel sequencing of millions and billions of DNA molecules, greatly increasing the scale and resolution of the analyzes. Despite being a relatively new technique, the use of NGS may correspond to a great tool for analysis of complete genomes, allowing the analysis of different drug targets.

All the studies that analyzed codon 200 of *A*. *lumbricoides* carried out genotyping of adult worms and/or egg pools [7,8,10]. Our study is the first to standardize and apply this technique to codon 200 on an expansive number of single eggs, showing that the technique is sensitive, specific and able to determine the genotype of a single egg. This finding is advantageous because egg pool genotyping may not match the most frequent genotypes in the analysis because the mixture of mutated homozygous eggs and wild-type homozygous eggs may erroneously result in heterozygous eggs. In addition, a given genotype may be surpassed in detriment of another genotype present more frequently in the analyzed pool.

The low number of searches in the public domain that have described the reduced effectiveness of anthelmintics does not provide real evidence of drug resistance among human STH. More phenotypic studies are needed to characterize populations of parasites that no longer respond to treatment. However, the presence of the mutation at codon 200 in the beta-tubulin isotype-1 of *A*. *lumbricoides* observed here may indicate an imminent problem for human health. In cases of recurrent treatment, the frequency of these alleles is likely to increase, as has been observed for veterinary parasites in recent decades. Because ascariasis is a neglected disease, the perpetuation of resistant parasites may be a problem, especially for poor populations, where parasite rates are higher, resulting in a cycle of infection, reinfection, and treatment failure.

## Supporting information

S1 Fig Chromatograms of mutated samples of *A*. *lumbricoides* at codon 200 in the beta-tubulin gene. Codon 200 is marked in black box.
(TIF)
