# Supplementary figures and images for: First identification of the benzimidazole resistance-associated F200Y SNP in the beta-tubulin gene in Ascaris lumbricoides
Source: PLoS One. 2019 Oct 17;14(10):e0224108. doi: 10.1371/journal.pone.0224108 (PMC6797262; doi:10.1371/journal.pone.0224108)

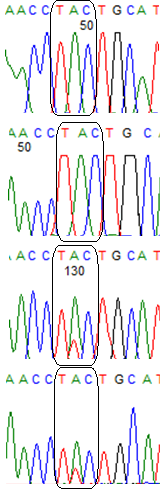

Supplement: S1 Fig — Codon 200 is marked in black box. (TIF) [file pone.0224108.s001.tif]
